# Tracing the Edible and Medicinal Plant *Pueraria montana* and Its Products in the Marketplace Yields Subspecies Level Distinction Using DNA Barcoding and DNA Metabarcoding

**DOI:** 10.3389/fphar.2020.00336

**Published:** 2020-03-20

**Authors:** Gaixia Zhang, Jinxin Liu, Mei Gao, Weijun Kong, Qing Zhao, Linchun Shi, Qiuling Wang

**Affiliations:** ^1^Institute of Medicinal Plant Development, Chinese Academy of Medical Sciences and Peking Union Medical College, Beijing, China; ^2^Hebei Key Laboratory of Study and Exploitation of Chinese Medicine, Chengde Medical University, Chengde, China

**Keywords:** *Pueraria montana*, DNA barcoding, DNA metabarcoding, identification, Gegen Powder, Yufeng Ningxin

## Abstract

Increased public awareness of nutritional and health issues has resulted in the increasing consumption of food and herbal products made from the root of *Pueraria montana* var. *lobata* (Willd.) Maesen & S. M. Almeida ex Sanjappa & Predeep (kudzu vine) and *P. montana* var. *thomsonii* (Benth.) M. R. Almeida. The famous herbal medicine Yufeng Ningxin, which is used to treat cardiovascular diseases, can be legally produced only using *P. montana* var. *lobata*. However, precise identification at the subspecies level is usually challenging when these products’ ingredients lose their morphological characteristics after deep processing. Here, six herbarium specimens, 21 expert-identified original plant samples, 30 raw material samples, 10 food products and 12 herbal products were collected to test the subspecies-level authentication abilities of ITS2 sequences. The results showed that ITS2 sequences can distinguish *P. montana* var. *lobata* from *P. montana* var. *thomsonii* with stable single nucleotide polymorphism (SNP) sites. A total of 93.3% of raw material samples were consistent with the markings on their labels, but only 50% of Gegen Powder samples were made from *P. montana* var. *lobata*. High-quality ITS2 sequences were successfully obtained from nine of the 12 herbal products using Sanger sequencing. Substitution and fungal contamination were detected in 3 herbal products by further DNA metabarcoding, even though thin-layer chromatography (TLC) and high-performance liquid chromatography (HPLC) tests verified that the products met existing quality standards. This study demonstrated that DNA barcoding is a powerful tool for the identification of *P. montana* var. *lobata* and *P. montana* var. *thomsonii* at the subspecies level, and we conclude that DNA barcodes can be broadly applied to trace the raw materials of food and herbal products.

## Introduction

*Pueraria montana* is a species in the plant family Fabaceae (Wu et al., 2010). It is native to China and has spread throughout many tropical regions, including those in Europe and the Americas. The biomass of its roots can account for up to 40% of total plant biomass and has been employed as a food and herbal medicine for thousands of years in regions of East and Southeast Asia (Bodner and Hymowitz, 2002). The food product has been called “longevity powder” or “Asian ginseng” due to its nutritive properties and high medicinal value (Cui et al., 2018). Three subspecies of *P. montana* are listed in the Flora of China (Wu et al., 2010): *P. montana* var. *lobata* (Willd.) Maesen & S. M. Almeida ex Sanjappa & Predeep (kudzu vine)*, P. montana* (Lour.) Merr. (syn. *P. montana* var. *montana)*, and *P. montana* var. *thomsonii* (Benth.) M. R. Almeida. The root, with or without periderm, of *P. montana* var. *lobata* is used as a type of herbal medicine, called Puerariae lobatae radix (also known as Gegen) in Chinese (C. P. Commission, 2015), European (E.P. Commission, 2014), Japanese (P.a.M.D.R.S. Society, and o. Japan, 2016), and Korean (K. MFDS, 2014) pharmacopoeias. The root of *P. montana* var. *thomsonii* is popularly used as a food and for extracting starch, although sometimes the root can be used as another herbal medicine called Puerariae thomsonii radix (also known as Fenge) in Chinese (C. P. Commission, 2015) and European (E.P. Commission, 2014) pharmacopoeias. The major active ingredient isolated from roots of the above two plants is puerarin (Zhou et al., 2014), which has a wide spectrum of pharmacological properties such as vasodilation, cardioprotection, neuroprotection, antioxidant activity, anticancer activity, anti-inflammatory activity, pain alleviation, bone formation promotion, alcohol intake inhibition, and insulin resistance attenuation. The official standard (C. P. Commission, 2015) for the puerarin content in Puerariae thomsonii radix (Fenge) is no less than 0.3%, whereas that in Puerariae lobatae radix (Gegen) is no less than 2.4%. The roots of the two plants have different functions, and Puerariae thomsonii radix (Fenge) is often substituted for Puerariae lobatae radix (Gegen) due to ecological cost.

*P. montana* var. *lobata* and *P. montana* var. *thomsonii* are listed in the directory of Chinese edible and medicinal homologous species, which means that products made from these species can be considered ordinary food in markets. The most commonly available food made from the root of *P. montana* var. *lobata* or *P. montana* var. *thomsonii* is Gegen Powder. Notably, although this food is called Gegen Powder, it is made from the powder of *P. montana* var. *thomsonii* root in most cases because this root produces more starch and costs less than the root of *P. montana* var. *lobata*. The price of Gegen Power made from *P. montana* var. *lobata* roots is usually higher than that made from *P. montana* var. *thomsonii* roots. Gegen Powder is widely sold in supermarkets and food stores and has been claimed to promote good health and longevity. Many Gegen Power products do not specifically indicate which species the raw materials are made from. The most commonly known and used herbal product made from *P. montana* var. *lobata* root (Gegen) extract and powder is Yufeng Ningxin (C. P. Commission, 2015), which is a famous traditional Chinese patent medicine listed in the “Catalogue of Drugs for Basic National Medical Insurance and Countermeasures (2019 version)”. Yufeng Ningxin can relieve spasms and promote blood circulation in the brain and coronary arteries, thus improving cardiovascular and cerebrovascular health. There are several different dosage forms made by different manufacturers, such as tablets, capsules, and pills. According to the 2015 edition of the Chinese Pharmacopeia (C. P. Commission, 2015), *P. montana* var. *lobata* root (Gegen) is the only legal raw material that can be used in Yufeng Ningxin, and it cannot be replaced by *P. montana* var. *thomsonii* root (Fenge). Unfortunately, consumers and patients cannot identify the actual materials contained in this herbal product because the morphological features of the subspecies are typically lost when the raw materials are deeply processed.

Recently, DNA barcoding has gained considerable support as an efficient tool for the authentication of food and medicinal products due to a high-quality DNA barcoding reference database (Chen et al., 2014; Chen et al., 2017; Liu et al., 2017). The success of DNA barcoding has thus prompted the Chinese Pharmacopoeia Commission and the British Pharmacopoeia Commission to incorporate DNA barcoding into the method list for routine herbal detection. Here, we conducted a systematic study to evaluate the ability of DNA barcoding and DNA metabarcoding to differentiate the two subspecies of *P. montana* and authenticate the raw materials for their food and herbal products.

## Materials and Methods

### Herbarium Specimens, Expert-Identified Original Plant Samples, Raw Material Samples, and Food and Herbal Products

Six herbarium specimens of *P. montana* var. *lobata* (Willd). Maesen & S .M. Almeida ex Sanjappa & Predeep (HHAA0001-HHAA0003) and *P. montana* var. *thomsonii* (Benth). M. R. Almeida (HHAA0004-HHAA0006) were collected from the Beijing Medical Botanical Garden and from Tengxian in Guangxi Province. These herbarium specimens have been authenticated by Dr. Qiuling Wang and are deposited in the Institute of Medicinal Plant Development herbarium (herbarium code “IMD”, NYBG: https://www.nybg.org/). Twenty-one expert-identified original plant samples of *P. montana* var. *lobata* (HSYS0001-HSYS0015) and *P. montana* var. *thomsonii* (HSYS0016-HSYS0021) were collected from Nanning in Guangxi Province, Tonghua in Jilin Province, and Shangrao in Jiangxi Province. One certified raw material sample of Puerariae lobatae radix (Gegen, HSTR0001) was bought from the Beijing Tongrentang herbal store. All herbarium specimens, original plant samples and certified raw material samples were curated by Dr. Qiuling Wang from the Institute of Medicinal Plant Development, Chinese Academy of Medicinal Sciences. Yufeng Ningxin reference tablets were formulated in a laboratory according to the methods documented in the Chinese Pharmacopoeia using certified Puerariae lobatae radix (Gegen) raw materials. Thirty commercial raw material samples (HSMS0001–HSMS0030), 10 food product samples (HSFS0001–HSFS0010), and 12 herbal product samples from six pharmaceutical companies (HSZY1001–HSZY1012) were purchased from herbal markets, food stores, supermarkets, and drug stores. The dosage forms of Yufeng Ningxin included tablets, capsules, and pills. The sampling information and GenBank accession numbers for the above materials are provided in **Table S1**.

### Operating Procedures for DNA Barcoding**, Thin-Layer Chromatography (TLC), and High-Performance Liquid Chromatography (HPLC)**

#### DNA Extraction, PCR Amplification, Sanger Sequencing, and Data Analysis

Genomic DNA of leaves from the herbarium specimens, raw material samples, food products and herbal product samples was extracted according to previously published protocols (Chen et al., 2010; Xin et al., 2018). The ITS2 sequences were amplified using the primers ITS2F (5’-ATGCGATACTTGGTGTGAAT-3’) and ITS3R (5’-GACGCTTCTCCAGACTACAAT-3’). PCR amplicons were detected using gel electrophoresis and then sequenced using a 3730XL sequencer (Applied Biosystems, USA). CodonCode Aligner 8.0.2 (CodonCode Co., USA) was used for trace file assembly. A hidden Markov model (HMM)-based annotation method was used for ITS2 annotation to remove the 5.8S and 28S rRNA region sequences (Keller et al., 2009; Wheeler and Eddy, 2013). Multiple sequence alignment was accomplished using Muscle 3.8 (Edgar, 2004). Kimura-two-parameter (K2P) distances were calculated with PAUP 4.0 (Swofford, 2002), and neighbor-joining (NJ) tree construction was performed using MEGA X software (Kumar et al., 2018).

#### High-Throughput Sequencing (HTS) and Bioinformatics Analysis

To mark the amplicons obtained from different samples, 10-bp tags were designed and added to the 5’ end of the universal ITS2 primers (**Table S2**). PCR products with marked primers were purified with a QIAquick PCR purification kit (Qiagen Inc., Germany). After purification, the PCR products were quantified and analyzed using a Qubit fluorometer (Thermo Fisher Scientific, Inc., USA) and an Agilent 2100 Bioanalyzer (Agilent Technologies, Inc., USA). The Ion Proton system (Thermo Fisher Scientific, Inc., USA) was employed to sequence these ITS2 amplicons. The raw sequencing reads in.fastq format were stored in the Sequence Read Archive (SRA) database with accession number SRR9822010.

Sequence reads for each sample were separated according to the 10-bp tags with a previously described Python script (Song et al., 2012). Low-quality reads with a minimum length < 300 bp and average quality value (QV) < 30 were removed. Target ITS2 loci were annotated using an HMM-based method as described by Keller et al. (Keller et al., 2009; Wheeler and Eddy, 2013). Unique sequences were obtained using a Python script (**File S1**), and unique sequences with more than 10 repetitions were retained. Species identification was implemented by searching the TCM barcode database (Chen et al., 2014).

TLC and HPLC analyses were performed according to procedures described in the Chinese Pharmacopoeia, 2015 edition (C. P. Commission, 2015).

## Results

### Intra- and Intersubspecies Variation in *Pueraria montana* var. *lobata* and *Pueraria montana* var. *thomsonii*

**Figure 1:**
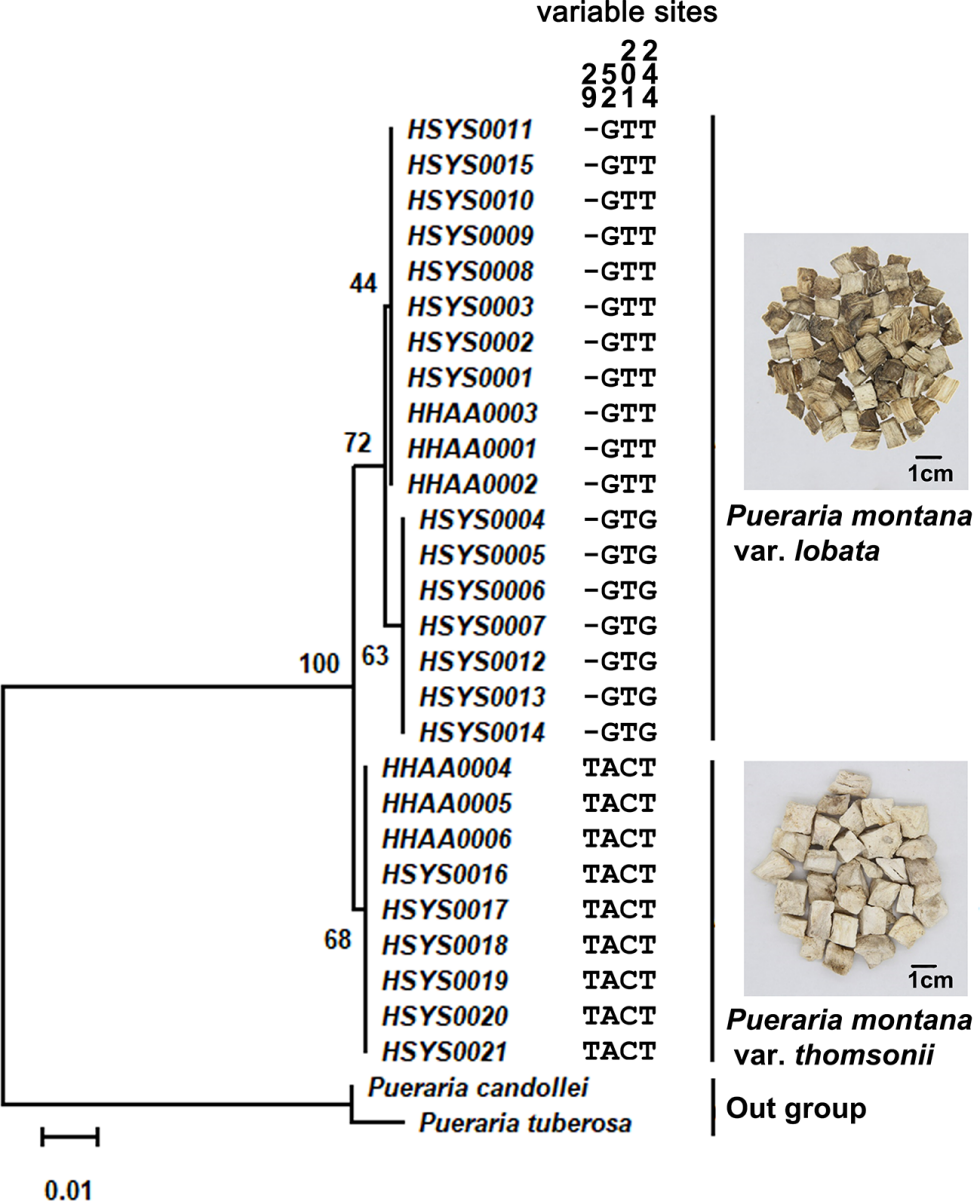
The NJ tree and sequence variable sites between *Pueraria montana* var. *lobata* and *Pueraria montana* var. *thomsonii*. The number of bootstrap replicates was 2000.

The ITS2 sequence lengths of *P. montana* var. *lobata* and *P. montana* var. *thomsonii* were 246 bp and 247 bp, respectively. No single nucleotide polymorphism (SNP) sites were found in the sequence of *P. montana* var. *thomsonii*, whereas one SNP site was found in the sequence of *P. montana* var. *lobata* (at 244 bp: T/G). One insertion/deletion variation was found at 29 bp (T/-), and three SNPs at 52 bp (A/G), 201 bp (C/T), and 244 bp (T/G) were found between the sequences of P. montana var. thomsonii and *P. montana* var. *lobata*. The maximum intraspecific distances of *P. montana* var. *lobata* and *P. montana* var. *thomsonii* were 0.0021 and 0, respectively, whereas the minimum interspecific distances for the two species was 0.0098. Therefore, a barcoding gap was present between the two subspecies because the minimum interspecific distance was greater than their maximum intraspecific distances. The ITS2 sequences of Pueraria tuberosa (GQ892046) and Pueraria candollei (LC424315) were downloaded from GenBank and chosen as the outgroup for NJ tree construction. The *P. montana* var. *lobata* and *P. montana* var. *thomsonii* samples clustered onto separate branches in the NJ tree and thus could be clearly distinguished (**Figure 1**). And, the sequence alignment profile between *P. montana* var. *lobata* and *P. montana* var. *thomsonii* was shown in **Figure S1**.

### Species Verification of Raw Material Samples of Puerariae lobatae radix (Gegen) and Puerariae thomsonii radix (Fenge) Purchased From Herbal Markets and Drug Stores by DNA Barcoding

High-quality DNA was easily obtained from all 30 raw material samples by extending the incubation time in the 56°C water bath to 8 *h* or overnight. PCR amplification was successfully performed, and all PCR products generated high-quality bidirectional Sanger sequencing chromatograms. All 30 raw material samples were successfully identified at the subspecies level by searching the TCM barcode database based on sequence similarity (**Table 1**). Among the 21 raw material samples labeled as Puerariae lobatae radix (Gegen), two were identified as Puerariae thomsonii radix (Fenge), and the remaining samples were confirmed to be Puerariae lobatae radix (Gegen). Among the 9 raw material samples labeled as Puerariae thomsonii radix (Fenge), all were verified to be Puerariae thomsonii radix (Fenge).

**Table 1:** Species verification of raw material samples of Puerariae lobatae radix (Gegen) and Puerariae thomsonii radix (Fenge) purchased from herbal markets by DNA barcoding.

Sample ID	Species listed on the label	Species identified by DNA barcoding
HSMS0001	*Pueraria montana* var. *lobata*	*Pueraria montana* var. *lobata*
HSMS0002	*Pueraria montana* var. *lobata*	*Pueraria montana* var. *lobata*
HSMS0003	*Pueraria montana* var. *lobata*	*Pueraria montana* var. *lobata*
HSMS0004	*Pueraria montana* var. *lobata*	*Pueraria montana* var. *lobata*
HSMS0005	*Pueraria montana* var. *lobata*	*Pueraria montana* var. *lobata*
HSMS0006	*Pueraria montana* var. *lobata*	*Pueraria montana* var. *lobata*
HSMS0007	*Pueraria montana* var. *lobata*	*Pueraria montana* var. *lobata*
HSMS0008	*Pueraria montana* var. *lobata*	*Pueraria montana* var. *lobata*
HSMS0009	*Pueraria montana* var. *lobata*	*Pueraria montana* var. *lobata*
HSMS0010	*Pueraria montana* var. *lobata*	*Pueraria montana* var. *lobata*
HSMS0011	*Pueraria montana* var. *lobata*	*Pueraria montana* var. *lobata*
HSMS0012	*Pueraria montana* var. *lobata*	*Pueraria montana* var. *lobata*
HSMS0013	*Pueraria montana* var. *lobata*	*Pueraria montana* var. *lobata*
HSMS0014	*Pueraria montana* var. *lobata*	*Pueraria montana* var. *lobata*
HSMS0015	*Pueraria montana* var. *lobata*	*Pueraria montana* var. *lobata*
HSMS0016	*Pueraria montana* var. *lobata*	*Pueraria montana* var. *lobata*
HSMS0017	*Pueraria montana* var. *lobata*	*Pueraria montana* var. *lobata*
HSMS0018	*Pueraria montana* var. *lobata*	*Pueraria montana* var. *lobata*
HSMS0019	*Pueraria montana* var. *lobata*	*Pueraria montana* var. *lobata*
HSMS0020	*Pueraria montana* var. *thomsonii*	*Pueraria montana* var. *thomsonii*
HSMS0021	*Pueraria montana* var. *thomsonii*	*Pueraria montana* var. *thomsonii*
HSMS0022	*Pueraria montana* var. *thomsonii*	*Pueraria montana* var. *thomsonii*
HSMS0023	*Pueraria montana* var. *thomsonii*	*Pueraria montana* var. *thomsonii*
HSMS0024	*Pueraria montana* var. *thomsonii*	*Pueraria montana* var. *thomsonii*
HSMS0025	*Pueraria montana* var. *thomsonii*	*Pueraria montana* var. *thomsonii*
HSMS0026	*Pueraria montana* var. *thomsonii*	*Pueraria montana* var. *thomsonii*
HSMS0027	*Pueraria montana* var. *thomsonii*	*Pueraria montana* var. *thomsonii*
HSMS0028	*Pueraria montana* var. *thomsonii*	*Pueraria montana* var. *thomsonii*
HSMS0029^$^	*Pueraria montana* var. *lobata*	*Pueraria montana* var. *thomsonii*
HSMS0030^$^	*Pueraria montana* var. *lobata*	*Pueraria montana* var. *thomsonii*

^$^Species has been mislabeled.

### Species Detection of Food Products Collected From Food Stores and Supermarkets by DNA Barcoding

**Figure 2:**
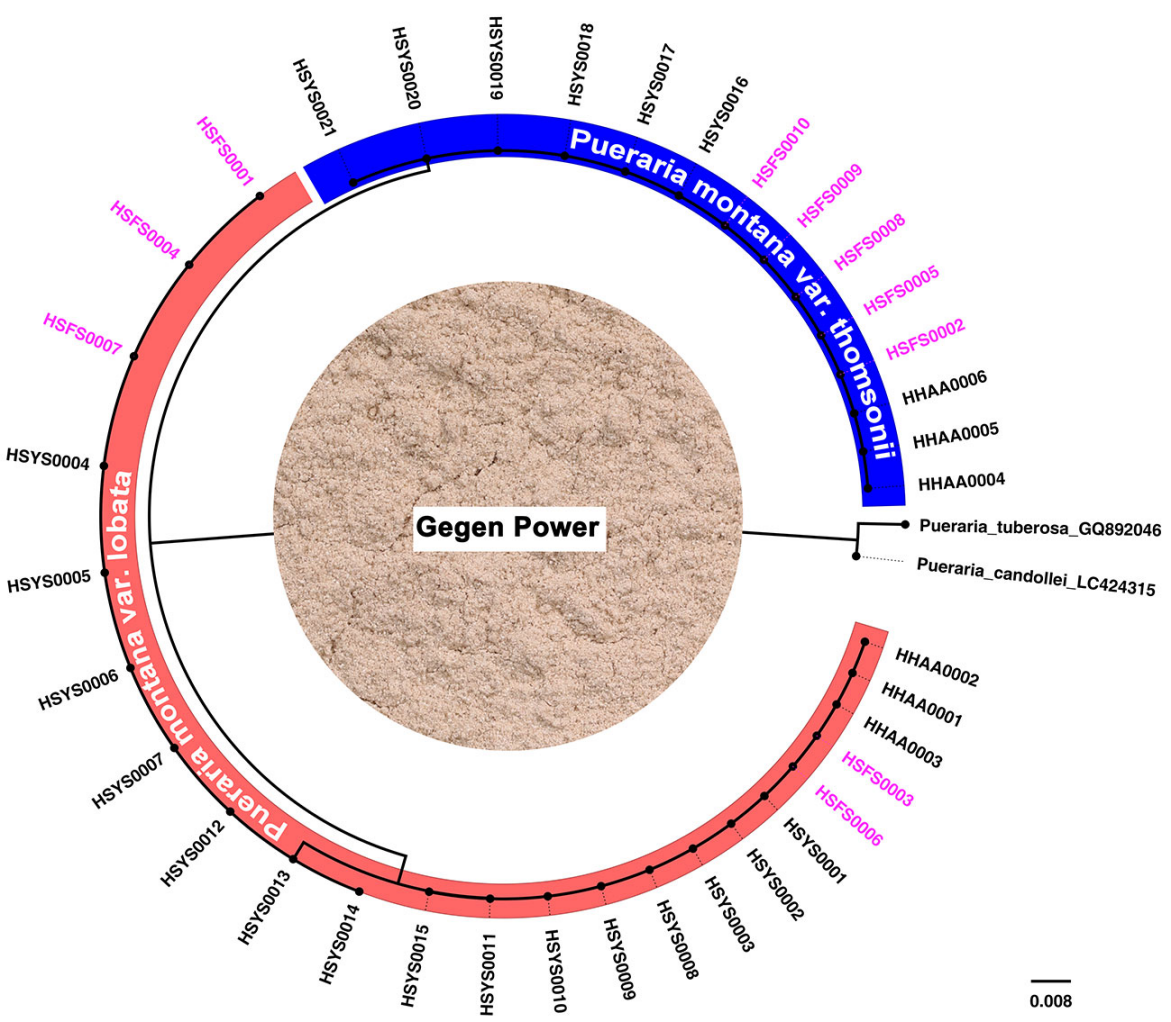
Species detection of food products by DNA barcoding. The subtree highlighted in blue is the *Pueraria montana* var. *thomsonii* group, and the subtree highlighted in red is the *Pueraria montana* var. *lobata* group. The picture in the middle of the figure shows Gegen Power. The labels in pink indicate samples of food products, and the other labels in black indicate reference specimens of *P. montana* var. *lobata* and *P. montana* var. *thomsonii*.

Gegen Powder is the main food product made from *P. montana* var. *lobata* or *P. montana* var. *thomsonii* roots. Most Gegen Powder available in the marketplace does not indicate whether the ingredients include *P. montana* var. *lobata* or *P. montana* var. *thomsonii*. Two kinds of processed Gegen Powder are currently available in the marketplace. One is made by first removing the root periderm and grinding the root without the periderm into powder, and the other product is made from starch extracted from the root after grinding. ITS2 sequences were successfully obtained for the first type of Gegen Powder but not for the second type of Gegen Powder. For the 10 food products belonging to the first type of Gegen Powder, the DNA barcoding identification results showed that the raw materials of five products included *P. montana* var. *lobata* root, and the other five products contained *P. montana* var. *thomsonii* root (**Figure 2**).

### Analysis of the Raw Materials Included in Yufeng Ningxin Herbal Products

**Figure 3:**
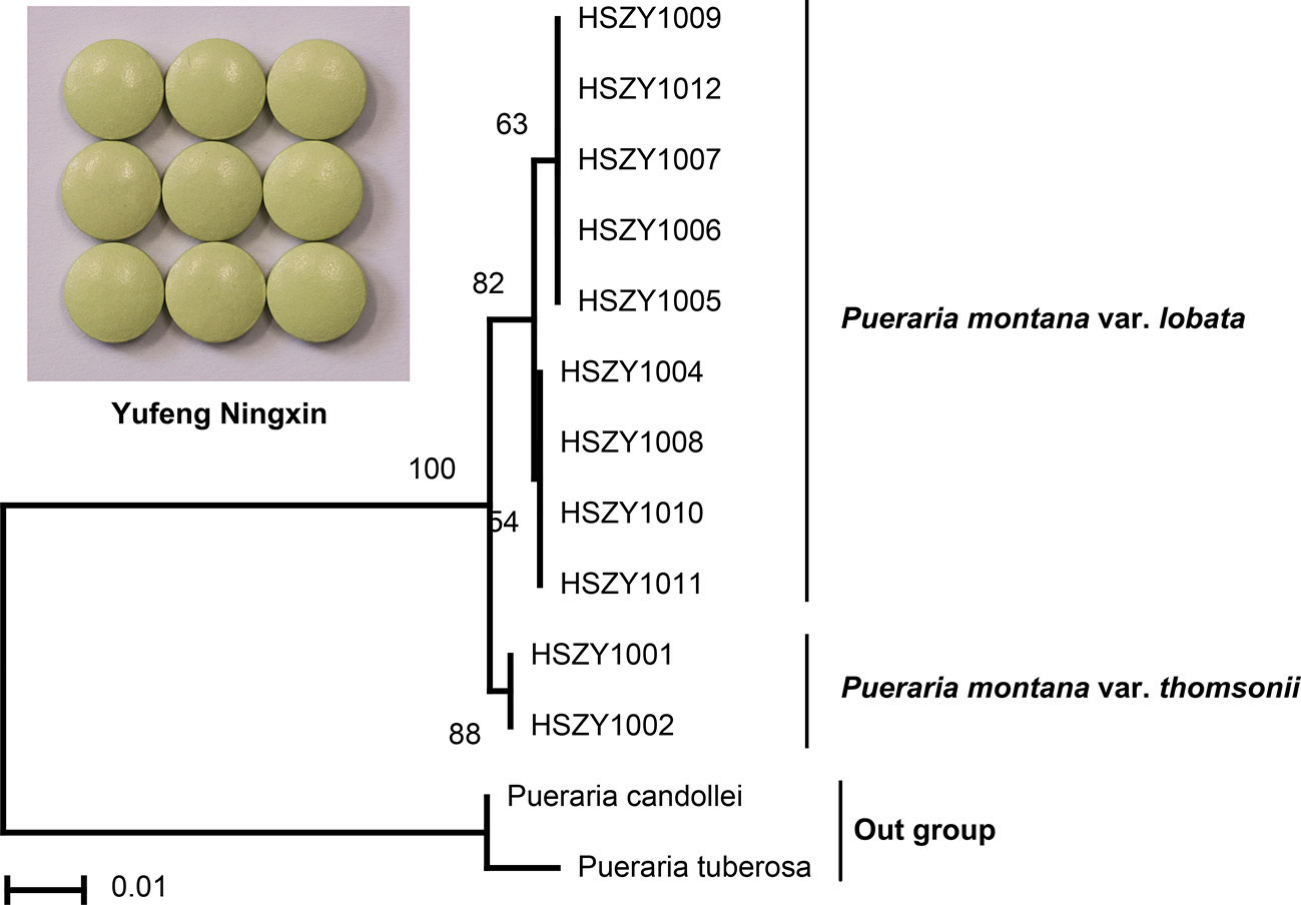
Species detection of Yufeng Ningxin herbal product samples by DNA barcoding.

The Puerariae lobatae radix (Gegen) raw material bought from the Beijing Tongrentang herbal store was first authenticated by DNA barcoding and then used to produce the reference Yufeng Ningxin herbal product in the laboratory for a pilot study (**Figure S2**). DNA extraction results indicated that high-quality DNA could be obtained from the Yufeng Ningxin reference, and relatively low-quality DNA was yielded from 12 commercial Yufeng Ningxin products, including tablets, capsules and pills from six manufacturers. PCR amplification and gel electrophoresis were successfully performed for the laboratory-produced Yufeng Ningxin reference and the 12 commercial Yufeng Ningxin products. The Sanger sequencing results could be divided into three types: type A (**Figure S3A**), where the product was sequenced successfully with high-quality bidirectional sequencing; type B (**Figure S3B**), where the product was suitable for bidirectional sequencing, with minimal manual editing of sequences required due to some heterozygous bases with shifting peaks; and type C (**Figure S3C**), where the product had unclear nested peaks and thus could not provide accurate and reliable DNA barcodes. The numbers of samples with type A, B, and C Sanger sequencing results were nine, two, and one, respectively. For the 11 commercial Yufeng Ningxin herbal products classified according to sequencing results as type A and type B, two were verified to mainly contain *P. montana* var. *thomsonii* root as a raw material (**Figure 3**).

### Substitution and Fungal Contamination in Yufeng Ningxin Herbal Products

To determine the substitution and contamination of herbal products, DNA metabarcoding was employed for three samples with the above three types of Sanger sequencing results. HSZY1006, HSZY1009, and HSZY1003 were selected to represent samples whose sequencing results were of type A, B, and C, respectively. The *P. montana* var. *thomsonii* subspecies was detected in the HSZY1003 commercial Yufeng Ningxin sample. Importantly, a certain number of reads for *Rosa* spp. were found in HSZY1009, and a large number of reads for *Hordeum vulgare* were detected in sample HSZY1003. In addition, fungal contamination was observed in all three commercial Yufeng Ningxin samples; in particular, a large proportion of species in the *Aspergillus* genus was detected. The exact number of reads and proportions indicating species composition in the three commercial samples are presented in **Table S3**. Finally, a pair of specific primer regions were designed to amplify sequences of the genus *Pueraria*, and the quality of the Sanger sequencing results was significantly improved (**Figure S4**).

### Chemical Testing of the Yufeng Ningxin Herbal Product Samples *via* TLC and HPLC

To test whether the Yufeng Ningxin commercial herbal product samples met current quality control standards, chemical tests using TLC and HPLC were performed. According to the TLC method in the Chinese Pharmacopoeia, the laboratory-produced Yufeng Ningxin reference had the same fluorescence spots as the reference standards at the corresponding position. All commercial specimens showed similar results in subsequent tests and were consistent with the laboratory-produced Yufeng Ningxin reference (**Figure S5**). According to the HPLC method in the Chinese Pharmacopoeia, Yufeng Ningxin tablets and capsules must contain no less than 13.0 mg and 20.0 mg of puerarin (C21H20O9), respectively. The values measured for the Yufeng Ningxin reference tablets and all commercial Yufeng Ningxin samples were all above the required standard (**Figure S6**, **Table S4**).

## Discussion

Due to increasing health awareness, the consumption of food products made from edible and medicinal species is rapidly increasing worldwide (Phan et al., 2017). From 2008 to 2017, the demand for *P. montana* root doubled to nearly 10,000 tons per year, and most of the added demand was due to the production of food products. However, the subspecies used to manufacture food products, such as Gegen Powder, is usually not indicated, while the same daily consumption is recommended regardless of the subspecies used. This has potential health risks because *P. montana* var. *lobata* root (Gegen) contains approximately eight times the amount of puerarin contained in *P. montana* var. *thomsonii* (Fenge) root. Recently, food safety concerns related to species authentication increased following news reports that a considerable number of fish labeled as salmon in China were actually rainbow trout. Fortunately, DNA barcoding has been verified to be a powerful and popular tool for the species authentication of food products (Xin et al., 2013), especially some highly processed food products. Wong et al. (Wong and Hanner, 2008) employed DNA barcoding to detect market substitution in North American seafood and found that 25% of the samples were potentially mislabeled. Here, we demonstrated that DNA barcoding technology is a powerful tool that can successfully detect the raw materials used to make Gegen Powder at the subspecies level. This technique will help improve the quality of this food and ultimately reduce the consumer safety risks associated with this type of food product.

TLC and HPLC methods were used under the guidance of the Chinese Pharmacopoeia for quality control of the herbal product Yufeng Ningxin. However, although TLC and HPLC tests verified that all of the commercial Yufeng Ningxin samples conformed to official standards, substitution was detected when employing DNA barcoding technology. Therefore, the currently used quality control method can classify herbal products as meeting official standards despite the substitution of raw materials. Therefore, we suggest that the DNA barcoding approach employed in this study be incorporated into currently used TLC and HPLC methods to precisely control the quality of herbal products. Incidentally, although an accurate species composition can be obtained *via* the DNA barcoding approach with HTS, this approach is not necessary in most instances, especially when the goal is simply to determine if there is species substitution. The Sanger sequencing results for herbal products with no raw material substitution or contamination should always be classified as type A. Type B and type C classification of samples may indicate a small or large amount of raw material substitution or contamination. This empirical classification method used to roughly judge whether there is substitution or contamination according to divergence in sequencing quality due to SNP sites has been thoroughly verified by Chen et al. (Chen et al., 2013).

The safety and efficacy of traditional patent medicines are controversial when unlabeled species are mixed into the formulation (Newmaster et al., 2013). Yufeng Ningxin is a single-biological-ingredient patent preparation that should contain only *P. montana* var. *lobata*, but substitution and contamination were found in this study. *P. montana* var. *thomsonii* was the main material used for substitution, and there is no clinical evidence to support the use of *P. montana* var. *thomsonii* root to make Yufeng Ningxin. In addition, the detection of fungi in herbal products is an alarming issue in the production and clinical application of patent medicines (Xin et al., 2018). Some species of fungi can produce secondary metabolites and mycotoxins (Sweeney and Dobson, 1998), especially when the raw materials are stored in a hot and humid environment. In this study, species of the genus *Aspergillus* were detected, and these species have the potential to produce aflatoxins (Sweeney and Dobson, 1998), which are causative agents of hepatocellular carcinoma. Unexpectedly, reads of *H. vulgare* were detected in one of the Yufeng Ningxin herbal product samples. We initially suspected that it was an experimental contaminant such as an aerosol, but when we designed a pair of specific primer regions to amplify the sequence of *H. vulgare* in this sample, the results showed that there were indeed sequences of *H. vulgare* in the sample. Therefore, one possible explanation for the detection of *H. vulgare* is contamination *via* a shared production line.

## Conclusion

The present study established a precise subspecies level identification method for food and herbal products made from the root of *Pueraria montana* var. *lobata* (kudzu vine) and *P. montana* var. *thomsonii* based on DNA barcoding method. Substitution has been found though TLC and HPLC tests verified that all of the commercial Yufeng Ningxin samples conformed to current official standards. It is suggested that the DNA barcoding method should be added to the existing quality control methods to trace the raw materials of food and herbal products, and then reducing the potential risk to public health.

## Data Availability Statement

The sequence data has been submitted to GenBank and their accession numbers are: MN202502, MN202503, MN202504, MN202556, MN202557, MN202558, MN202505, MN202576, MN202506, MN202507, MN202554, MN202508, MN202509, MN202555, MN202577, MN202578, MN202510, MN202511, MN202512, MN202513, MN202514, MN202515, MN202516, MN202517, MN202518, MN202519, MN202520, MN202521, MN202522, MN202523, MN202524, MN202525, MN202526, MN202527, MN202528, MN202565, MN202570, MN202562, MN202561, MN202559, MN202563, MN202560, MN202564, MN202568, MN202566, MN202567, MN202529, MN202530, MN202531, MN202532, MN202533, MN202534, MN202535, MN202536, MN202537, MN202538, MN202539, MN202540, MN202541, MN202542, MN202543, MN202544, MN202569, MN202571, MN202572, MN202575, MN202573, MN202574, MN202579, MN202580, MN202545, MN202546, MN202547, MN202548, MN202549, MN202550, MN202551, MN202552, MN202553.

## Author Contributions

LS and QW designed researches. GZ, JL, MG, QZ, and WK performed the experiment. GZ, LS, and JL analyzed the data. GZ, LS, QW, and JL wrote the paper. LS and JL revised the paper.

## Funding

This work was supported by the Chinese Academy of Medical Sciences, Innovation Funds for Medical Sciences (CIFMS) [2016-I2M-3-016, 2016-I2M-2-003], National Natural Science Foundation of China (81703659), Beijing Municipal Natural Science Foundation (7202136), and Major Research and Development Projects of Sichuan Science and Technology Plan Projects (2019YFS0017).

## Conflict of Interest

The authors declare that the research was conducted in the absence of any commercial or financial relationships that could be construed as a potential conflict of interest.
