# Supplementary material for: Tracing the Edible and Medicinal Plant Pueraria montana and Its Products in the Marketplace Yields Subspecies Level Distinction Using DNA Barcoding and DNA Metabarcoding
Source: Front Pharmacol. 2020 Mar 20;11:336. doi: 10.3389/fphar.2020.00336 (PMC7098995; doi:10.3389/fphar.2020.00336)
Supplement: Supplementary file 1 [file DataSheet_1.pdf]

Supplementary Tables

Table S1 The sampling information and GenBank accession numbers for herbarium specimens, expert-identified original plant samples, raw material samples, food products, and herbal products.

Sample ID	Species	Location	Accession No.
HHAA0001	*Pueraria montana* var. *lobata*	Beijing Medical Botanical Garden	MN202502
HHAA0002	*Pueraria montana* var. *lobata*	Beijing Medical Botanical Garden	MN202503
HHAA0003	*Pueraria montana* var. *lobata*	Beijing Medical Botanical Garden	MN202504
HHAA0004	*Pueraria montana* var. *thomsonii*	Tengxian, Guangxi	MN202556
HHAA0005	*Pueraria montana* var. *thomsonii*	Tengxian, Guangxi	MN202557
HHAA0006	*Pueraria montana* var. *thomsonii*	Tengxian, Guangxi	MN202558
HSFS0001	*Pueraria montana* var. *lobata*	Food store	MN202505
HSFS0002	*Pueraria montana* var. *thomsonii*	Food store	MN202576
HSFS0003	*Pueraria montana* var. *lobata*	Food store	MN202506
HSFS0004	*Pueraria montana* var. *lobata*	Food store	MN202507
HSFS0005	*Pueraria montana* var. *thomsonii*	Food store	MN202554
HSFS0006	*Pueraria montana* var. *lobata*	Food store	MN202508
HSFS0007	*Pueraria montana* var. *lobata*	Food store	MN202509
HSFS0008	*Pueraria montana* var. *thomsonii*	Food store	MN202555
HSFS0009	*Pueraria montana* var. *thomsonii*	Food store	MN202577
HSFS0010	*Pueraria montana* var. *thomsonii*	Food store	MN202578
HSMS0001	*Pueraria montana* var. *lobata*	Herbal market	MN202510
HSMS0002	*Pueraria montana* var. *lobata*	Herbal market	MN202511
HSMS0003	*Pueraria montana* var. *lobata*	Herbal market	MN202512
HSMS0004	*Pueraria montana* var. *lobata*	Herbal market	MN202513
HSMS0005	*Pueraria montana* var. *lobata*	Herbal market	MN202514
HSMS0006	*Pueraria montana* var. *lobata*	Herbal market	MN202515
HSMS0007	*Pueraria montana* var. *lobata*	Herbal market	MN202516
HSMS0008	*Pueraria montana* var. *lobata*	Herbal market	MN202517
HSMS0009	*Pueraria montana* var. *lobata*	Herbal market	MN202518
HSMS0010	*Pueraria montana* var. *lobata*	Herbal market	MN202519
HSMS0011	*Pueraria montana* var. *lobata*	Herbal market	MN202520
HSMS0012	*Pueraria montana* var. *lobata*	Herbal market	MN202521
HSMS0013	*Pueraria montana* var. *lobata*	Herbal market	MN202522
HSMS0014	*Pueraria montana* var. *lobata*	Herbal market	MN202523
HSMS0015	*Pueraria montana* var. *lobata*	Herbal market	MN202524

HSMS0016	*Pueraria montana* var. *lobata*	Herbal market	MN202525
HSMS0017	*Pueraria montana* var. *lobata*	Herbal market	MN202526
HSMS0018	*Pueraria montana* var. *lobata*	Herbal market	MN202527
HSMS0019	*Pueraria montana* var. *lobata*	Herbal market	MN202528
HSMS0020	*Pueraria montana* var. *thomsonii*	Herbal market	MN202565
HSMS0021	*Pueraria montana* var. *thomsonii*	Herbal market	MN202570
HSMS0022	*Pueraria montana* var. *thomsonii*	Herbal market	MN202562
HSMS0023	*Pueraria montana* var. *thomsonii*	Herbal market	MN202561
HSMS0024	*Pueraria montana* var. *thomsonii*	Herbal market	MN202559
HSMS0025	*Pueraria montana* var. *thomsonii*	Herbal market	MN202563
HSMS0026	*Pueraria montana* var. *thomsonii*	Herbal market	MN202560
HSMS0027	*Pueraria montana* var. *thomsonii*	Herbal market	MN202564
HSMS0028	*Pueraria montana* var. *thomsonii*	Herbal market	MN202568
HSMS0029	*Pueraria montana* var. *thomsonii*	Herbal market	MN202566
HSMS0030	*Pueraria montana* var. *thomsonii*	Herbal market	MN202567
HSTR0001	*Pueraria montana* var. *lobata*	Tongrentang, Beijing	MN202529
HSYS0001	*Pueraria montana* var. *lobata*	Xinyang, Henan	MN202530
HSYS0002	*Pueraria montana* var. *lobata*	Xinyang, Henan	MN202531
HSYS0003	*Pueraria montana* var. *lobata*	Xinyang, Henan	MN202532
HSYS0004	*Pueraria montana* var. *lobata*	Xinyang, Henan	MN202533
HSYS0005	*Pueraria montana* var. *lobata*	Xinyang, Henan	MN202534
HSYS0006	*Pueraria montana* var. *lobata*	Xinyang, Henan	MN202535
HSYS0007	*Pueraria montana* var. *lobata*	Xinyang, Henan	MN202536
HSYS0008	*Pueraria montana* var. *lobata*	Xinyang, Henan	MN202537
HSYS0009	*Pueraria montana* var. *lobata*	Xinyang, Henan	MN202538
HSYS0010	*Pueraria montana* var. *lobata*	Nanning, Guangxi	MN202539
HSYS0011	*Pueraria montana* var. *lobata*	Nanning, Guangxi	MN202540
HSYS0012	*Pueraria montana* var. *lobata*	Nanning, Guangxi	MN202541
HSYS0013	*Pueraria montana* var. *lobata*	Tonghua, Jilin	MN202542
HSYS0014	*Pueraria montana* var. *lobata*	Tonghua, Jilin	MN202543
HSYS0015	*Pueraria montana* var. *lobata*	Tonghua, Jilin	MN202544
HSYS0016	*Pueraria montana* var. *thomsonii*	Nanping, Fujian	MN202569
HSYS0017	*Pueraria montana* var. *thomsonii*	Nanping, Fujian	MN202571
HSYS0018	*Pueraria montana* var. *thomsonii*	Shangrao, Jiangxi	MN202572
HSYS0019	*Pueraria montana* var. *thomsonii*	Shangrao, Jiangxi	MN202575
HSYS0020	*Pueraria montana* var. *thomsonii*	Shangrao, Jiangxi	MN202573
HSYS0021	*Pueraria montana* var. *thomsonii*	Shangrao, Jiangxi	MN202574
HSZY1001	*Pueraria montana* var. *thomsonii*	Drug store	MN202579
HSZY1002	*Pueraria montana* var. *thomsonii*	Drug store	MN202580
HSZY1003	--	Drug store	--
HSZY1004	*Pueraria montana* var. *lobata*	Drug store	MN202545

HSZY1005	*Pueraria montana* var. *lobata*	Drug store	MN202546
HSZY1006	*Pueraria montana* var. *lobata*	Drug store	MN202547
HSZY1007	*Pueraria montana* var. *lobata*	Drug store	MN202548
HSZY1008	*Pueraria montana* var. *lobata*	Drug store	MN202549
HSZY1009	*Pueraria montana* var. *lobata*	Drug store	MN202550
HSZY1010	*Pueraria montana* var. *lobata*	Drug store	MN202551
HSZY1011	*Pueraria montana* var. *lobata*	Drug store	MN202552
HSZY1012	*Pueraria montana* var. *lobata*	Drug store	MN202553

Table S2 Ten-base pair tags added to the 5' end of the universal ITS2 primers for herbal product samples HSZY1006, HSZY1009, and HSZY1003.

Sample ID	Tag sequence	Forward primer with tag	Reverse primer with tag
HSZY1006	TCATCGAGTC	TCATCGAGTCGAGTCTT TGAACGCAAGTTG	TCATCGAGTCTCCTCC GCTTATTGATATG
HSZY1009	TCTGATCGAG	TCTGATCGAGGAGTCTT TGAACGCAAGTTG	TCTGATCGAGTCCTCC GCTTATTGATATG
HSZY1003	TGACCATTCG	TGACCATTCGGAGTCTT TGAACGCAAGTTG	TGACCATTCGTCCTCC GCTTATTGATATG

Table S3 The exact number of reads and proportions indicating species composition in herbal product samples HSZY1006, HSZY1009, and HSZY1003.

**Sample ID: HSZY1006**		
**Species**	**No. of reads**	**Proportion (%)**
Aspergillus ruber	120671	35.72
Pueraria montana var. lobata	76220	22.56
Mucor racemosus f. sphaerosporus	41122	12.17
Aspergillus vitricola	17199	5.09
Tausonia pullulans	11155	3.3
Cladosporium pseudocladosporioides	9943	2.94
Aspergillus niveoglaucus	9330	2.76
Cystofilobasidium infirmominiatum	8903	2.64
Penicillium brevicompactum	4514	1.34
Other spcies with less than 1% reads	38786	11.49
**Sample ID: HSZY1009**		
**Species**	**No. of reads**	**Proportion (%)**
Pueraria montana var. lobata	26902	33
Hansenia forbesii	24125	29.59
Mucor racemosus f. sphaerosporus	6777	8.31
Aspergillus ruber	5418	6.65
Astragalus mongholicus	4786	5.87

Rosa rugosa	2307	2.83
Rosa setigera	1286	1.58
Rosa blanda	938	1.15
Other spcies with less than 1% reads	8988	11.03

**Sample ID: HSZY1003**		
**Species**	**No. of reads**	**Proportion (%)**
Hordeum vulgare	438206	57.67
Cuscuta australis	96534	12.7
Hordeum vulgare subsp. spontaneum	64818	8.53
Rosa rugosa	21354	2.81
Aspergillus amstelodami	15336	2.02
Trollius membranostylis	14067	1.85
Codonopsis pilosula var. modesta	10559	1.39
Aspergillus flavus	10376	1.37
Vicia sativa	9595	1.26
Rhodiola crenulata	9175	1.21
Other spcies with less than 1% reads	69818	9.17

Table S4 Puerarin content in Yufeng Ningxin samples determined with HPLC technology.

**Sampe ID**	**Puerarin Content (n=3)**	
	**Mean（mg/unit[$]）**	**SD**
HSZY1001	18.87	0.3135
HSZY1002	19.45	0.1339
HSZY1003	22.34	0.1753
HSZY1004	12.98	0.0808
HSZY1005	22.43	0.0395
HSZY1006	23.88	0.1093
HSZY1007	32.63	0.4253
HSZY1008	29.09	0.2376
HSZY1009	26.39	0.1085
HSZY1010	18.89	0.1968
HSZY1011	18.06	0.1364
HSZY1012	22.54	0.076
Lab made reference sample	27.65	0.1395

[$] pill, tablet or capsule

Supplementary Figures

```
Pueraria_montana_var._lobata_HHAA0001     CACATCGTTA CCCCAACGCA AACAGACG-T CCCACACGAC GGCCGTTGCG[ 50]
Pueraria_montana_var._lobata_HHAA0002     .......... .......... ........-. .......... ..........[ 50]
Pueraria_montana_var._lobata_HHAA0003     .......... .......... ........-. .......... ..........[ 50]
Pueraria_montana_var._thomsonii_HHAA0004  .......... .......... ........T. .......... ..........[ 50]
Pueraria_montana_var._thomsonii_HHAA0005  .......... .......... ........T. .......... ..........[ 50]
Pueraria_montana_var._thomsonii_HHAA0006  .......... .......... ........T. .......... ..........[ 50]

Pueraria_montana_var._lobata_HHAA0001     TGGTAGGGTG CACGCTGACC TCCCGCGAGC GGCGTCTCGC GGTTGGTTGA[100]
Pueraria_montana_var._lobata_HHAA0002     .......... .......... .......... .......... ..........[100]
Pueraria_montana_var._lobata_HHAA0003     .......... .......... .......... .......... ..........[100]
Pueraria_montana_var._thomsonii_HHAA0004  .A........ .......... .......... .......... ..........[100]
Pueraria_montana_var._thomsonii_HHAA0005  .A........ .......... .......... .......... ..........[100]
Pueraria_montana_var._thomsonii_HHAA0006  .A........ .......... .......... .......... ..........[100]

Pueraria_montana_var._lobata_HHAA0001     AAATCGAGTT CGCGGCCGAG CACGCCGTGA TAAAATGGTG GATGAGCAAC[150]
Pueraria_montana_var._lobata_HHAA0002     .......... .......... .......... .......... ..........[150]
Pueraria_montana_var._lobata_HHAA0003     .......... .......... .......... .......... ..........[150]
Pueraria_montana_var._thomsonii_HHAA0004  .......... .......... .......... .......... ..........[150]
Pueraria_montana_var._thomsonii_HHAA0005  .......... .......... .......... .......... ..........[150]
Pueraria_montana_var._thomsonii_HHAA0006  .......... .......... .......... .......... ..........[150]

Pueraria_montana_var._lobata_HHAA0001     GCTCGAGACC AATCACGCGC TGCGACTCGG TCCGCGAAGG ACTCCCTGAT[200]
Pueraria_montana_var._lobata_HHAA0002     .......... .......... .......... .......... ..........[200]
Pueraria_montana_var._lobata_HHAA0003     .......... .......... .......... .......... ..........[200]
Pueraria_montana_var._thomsonii_HHAA0004  .......... .......... .......... .......... ..........[200]
Pueraria_montana_var._thomsonii_HHAA0005  .......... .......... .......... .......... ..........[200]
Pueraria_montana_var._thomsonii_HHAA0006  .......... .......... .......... .......... ..........[200]

Pueraria_montana_var._lobata_HHAA0001     TGATGACGAC CCTACAGTGC GCCTCCTCTC CGGAGACGCT CTCTACG[247]
Pueraria_montana_var._lobata_HHAA0002     .......... .......... .......... .......... .......[247]
Pueraria_montana_var._lobata_HHAA0003     .......... .......... .......... .......... .......[247]
Pueraria_montana_var._thomsonii_HHAA0004  C......... .......... .......... .......... .......[247]
Pueraria_montana_var._thomsonii_HHAA0005  C......... .......... .......... .......... .......[247]
Pueraria_montana_var._thomsonii_HHAA0006  C......... .......... .......... .......... .......[247]
```

Figure S1 The sequence alignment profile between *P. montana* var. *lobata* and *P. montana* var. *thomsonii*.

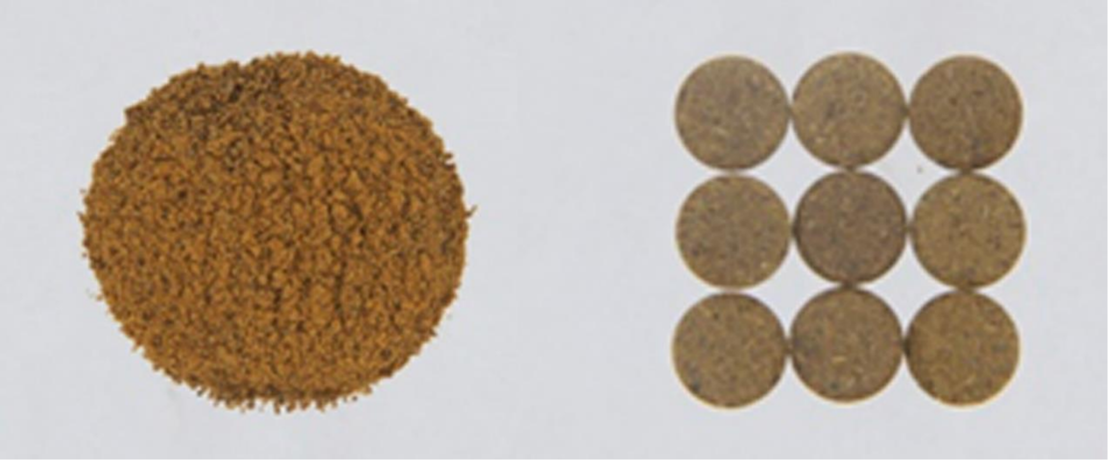

Figure S2 The reference Yufeng Ningxin herbal product made in the laboratory for a pilot study.

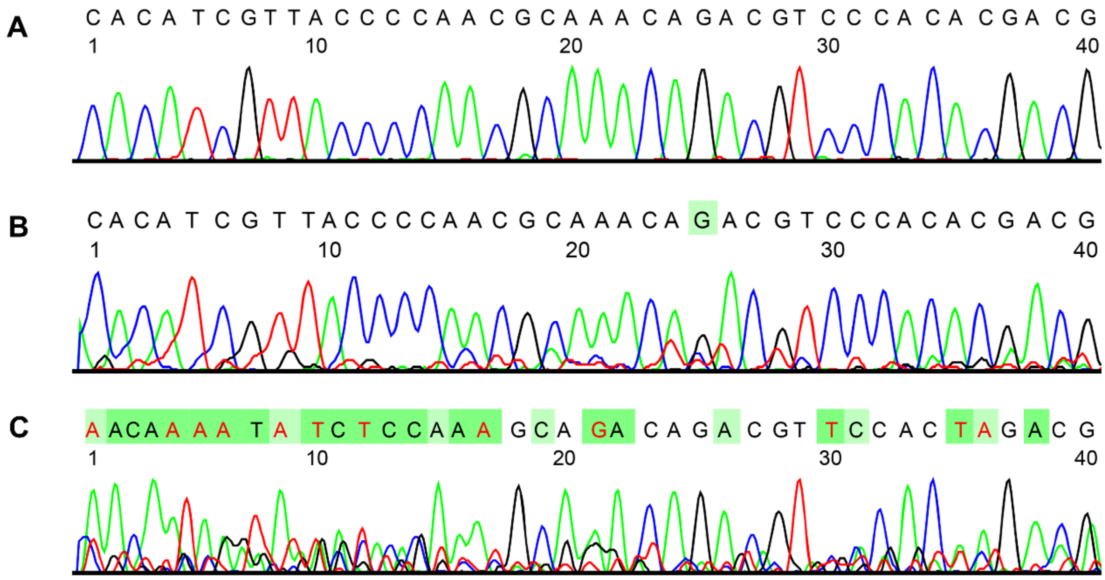

Figure S3 Sanger sequencing results for the commercial Yufeng Ningxin herbal product. A. High-quality bidirectional sequencing result, B. sequencing result with some heterozygous bases showing shifting peaks but that can be curated manually, C. sequencing result with many unclear nested peaks and without a suitable consensus sequence for species identification.

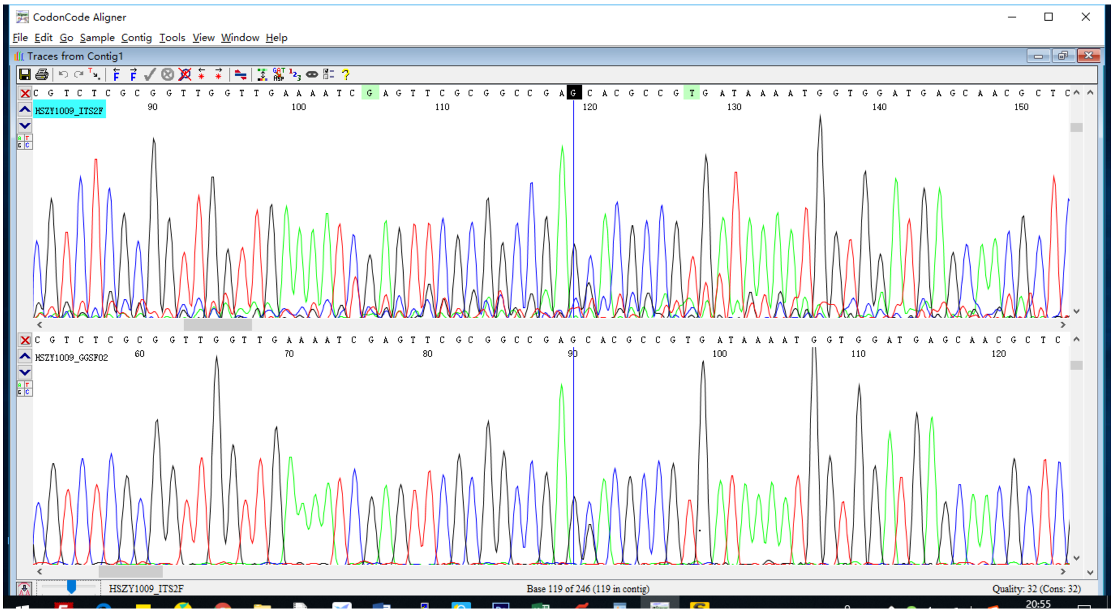

Figure S4 The quality of the Sanger sequencing results was significantly improved when specific primer pairs for the genus *Pueraria* were used. The upper part of the figure shows the trace file obtained using the universal forward primer ITS2F, whereas

the lower part of the figure shows the trace file obtained using the specific primer GGSF02.

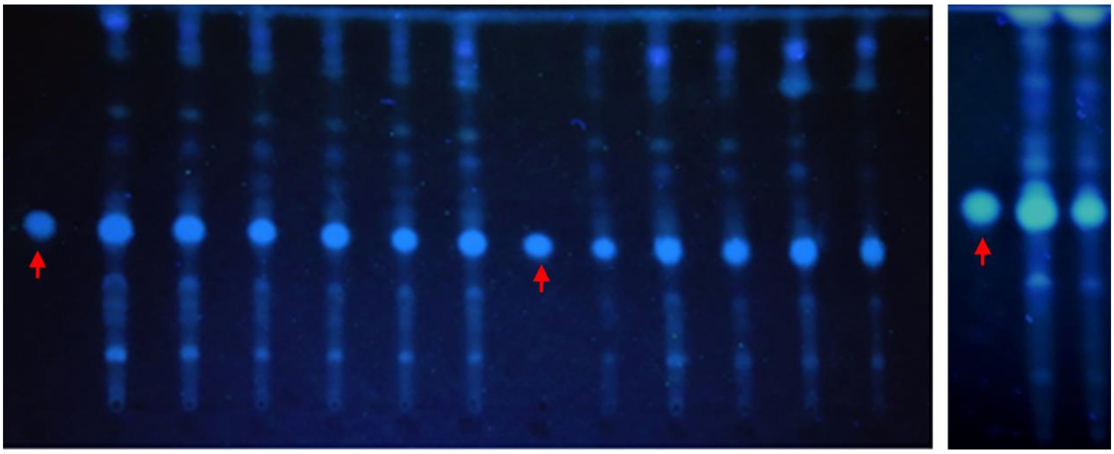

Figure S5 TLC chromatogram of the chemical reference standards, laboratory-made Yufeng Ningxin reference and commercial Yufeng Ningxin samples. The red arrow indicates the chemical reference standard of puerarin.

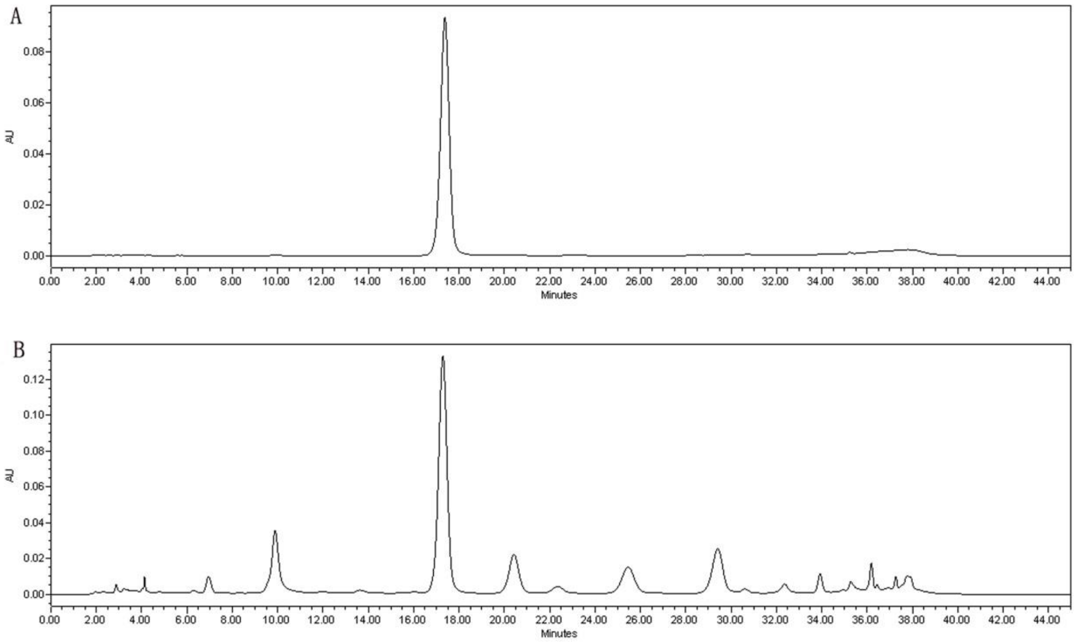

Figure S6 HPLC chromatogram of the puerarin chemical reference standard (A) and the Yufeng Ningxin sample (B).

Supplementary Files

File S1 Python script for obtaining unique sequences (findUniqSequence.py)

```
# -*- coding: UTF-8 -*-
import sys
import os
from Bio import SeqIO

#################################
print ("Usage: inputFile outputFile cutoff")

workfile = sys.argv[1] #inputfile with all sequences
out = open(sys.argv[2], "w") #outfile with unique sequences
cutoff = int(sys.argv[3])

##################################
D = {}
for record in SeqIO.parse(workfile, "fasta"):
	DNA = str(record.seq)
	if DNA in D.keys():
		D[DNA].append(record.id)
	else:
		D[DNA] = [record.id]

L = sorted(D.items(), key=lambda x:len(x[1]), reverse=True)
for it in L:
	if len(it[1]) >= cutoff:

out.write(">"+it[1][0]+"|"+str(len(it[1]))+"\n"+it[0]+"\n")
out.close()
```
